# Initial stability of cementless acetabular cups: press-fit and screw fixation interaction—an in vitro biomechanical study

**DOI:** 10.1007/s00590-014-1571-4

**Published:** 2014-11-25

**Authors:** Tomonori Tabata, Nobuhiro Kaku, Katsutoshi Hara, Hiroshi Tsumura

**Affiliations:** Department of Orthopaedic Surgery, Faculty of Medicine, Oita University, Oita, 879-5593 Japan

**Keywords:** Total hip arthroplasty, Cementless acetabular cup, Initial stability, Press-fit fixation, Screw fixation, Biomechanical study

## Abstract

**Background:**

Press-fit and screw fixation are important technical factors to achieve initial stability of a cementless acetabular cup for good clinical results of total hip arthroplasty. However, how these factors affect one another in initial cup fixation remains unclear. Therefore, this study aimed to evaluate the mutual influence between press-fit and screw fixation on initial cup stability.

**Methods:**

Foam bone was subjected to exact hemispherical-shape machining to diameters of 48, 48.5 and 49 mm. A compressive force was applied to ensure seating of a 48-mm-diameter acetabular cup in the foam bone prior to testing. Screws were inserted in six different conditions and tightened in a radial direction at the same torque strength. Then, the socket was rotated with a twist-testing machine, and the torque value at the start of axial rotation between the socket and the foam bone was measured under each screw condition.

**Results:**

The torque values for the 48-mm-diameter reaming were >20 N m higher than those for the 48.5- and 49-mm-diameter reaming in each screw condition, indicating that press-fit fixation is stronger than screw fixation. Meanwhile, torque values for the 48.5- and 49-mm-diameter reaming tended to increase with increasing the number of screws.

**Conclusions:**

According to our experiment, press-fit fixation of a cementless acetabular cup achieved rigid stability. Although the supplemental screws increased stability of the implant under good press-fit conditions, they showed little impact on whole-cup stability. In the case of insufficient press-fit fixation, cup stability depends on screw stability and increasing the number of additional screws increases cup stability.

## Introduction

Press-fit and screw fixation are important technical factors for achieving the initial stability of the acetabular cup in cementless total hip arthroplasty (THA). Press-fit fixation involves pressure bonding by differences in elasticity between the bone and the metal cup; the diameter of the cup is usually bigger than the reaming diameter of the acetabular bone. If the acetabular bone is very sclerotic and hard, or if the hemispherical dome created by reaming is smaller than necessary, it is difficult for the acetabular cup to contact the bottom of the bone bed since its pole cannot reach the predetermined depth. Under these conditions, a gap will be created between the cup and the bone. If the cup is hit to prevent creating a gap, it can lead to acetabular fracture around the cup [1]. Conversely, if the hemispherical dome created by reaming is larger than necessary, especially in older patients with osteoporosis, initial press-fit fixation cannot be expected to gain sufficient cup stability. However, screw fixation has broad utility and reliability in orthopedic surgery, and it has been widely used in osteosynthesis. Since an increasing number of screw holes in a cementless cup results in a decrease in the treated surface area for stimulating bone ingrowth, and also a greater possibility of developing backside wear of the polyethylene liner, it is not an effective solution for a good long-term clinical behavior of THA. In addition, because inserting a screw of the wrong length or in the wrong course risks vascular injury, it is improbable to leverage all the screw holes in clinical use.

Concerning both fixation techniques, some reports place emphasis on press-fit fixation [2–6], while others note that bone screws are very helpful aids in cup fixation [7–10]. However, those studies reported the benefits and limitations of press-fit and screw fixation independently. Clinically, the acetabular cup in cementless THA is fixed to the bone with a combination of press-fit and screw fixation, and both techniques influence one another, possibly increasing the initial strength of the whole cup. However, there has been no recent report detailing the relationship between both techniques in initial cup fixation. Therefore, the purpose of this study was to evaluate the mutual influence between press-fit and additional screw fixation on initial cup stability.

## Materials and methods

**Fig. 1:**
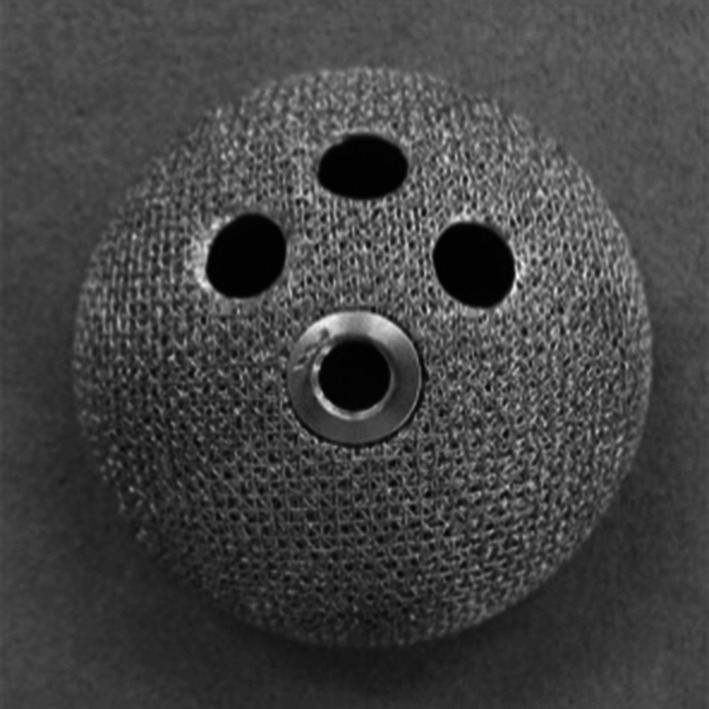
Titanium mesh-coated acetabular cup (Nakashima THA Cup; Nakashima Medical Co. Ltd, Okayama, Japan) with three screw holes and a diameter of 48 mm

A commercially available hemispherical acetabular cup (Nakashima THA Cup, Nakashima Medical Co. Ltd, Okayama, Japan) with three screw holes and an outer diameter of 48 mm was used in this study. Although the diameter of the cup has been notarized as 48 mm, the actual diameter was 48.4 mm (Fig. 1). The surface of the acetabular cup was treated with a titanium fiber mesh coating. The screws used were 20-mm long with a 6.5-mm outer diameter and a 3.1-mm inner diameter. A fresh-frozen human cadaveric pelvis would have been preferred for this biomechanical testing, but the necessary number of bones of the same quality, matched for sex, age, and size, could not be obtained in Japan. Consequently, we used foam bone in this study.

**Fig. 2:**
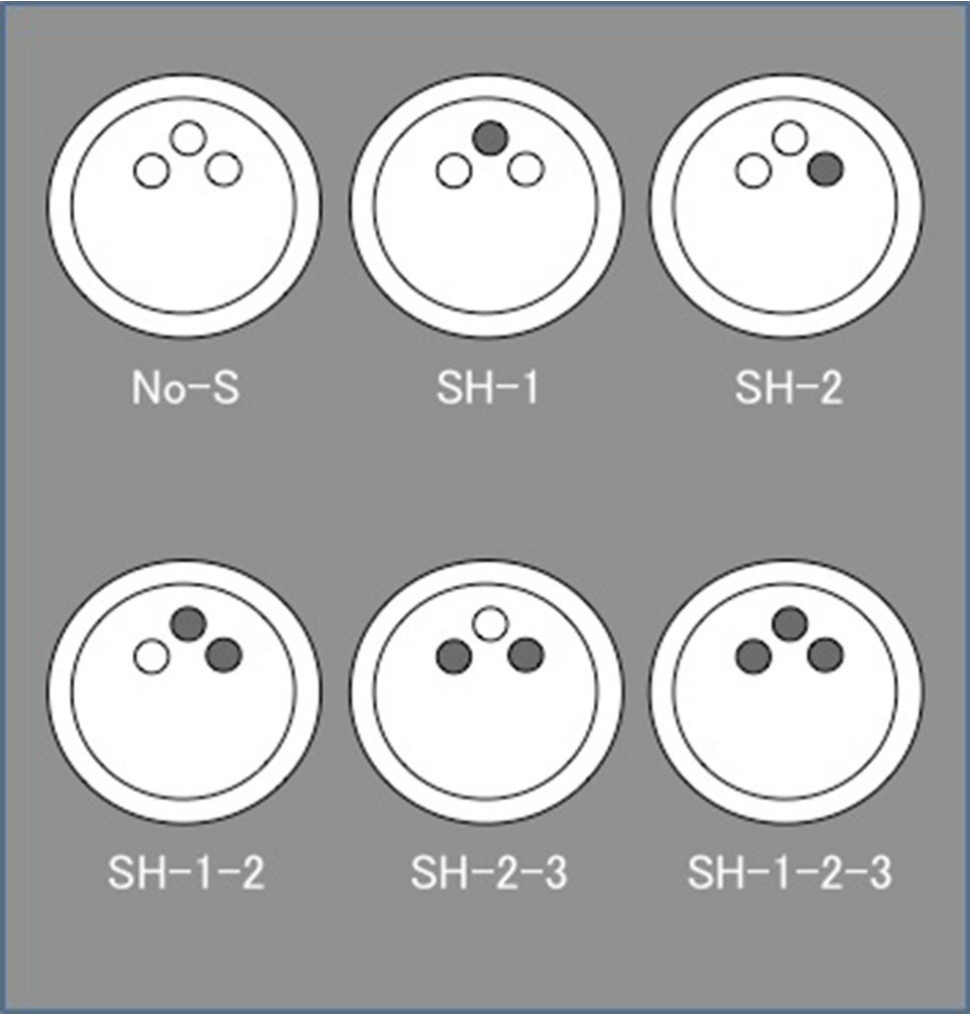
The screws are inserted in the following six conditions: no screw (No-S), one screw (SH-1 and SH-2), two screws (SH-1-2 and SH-2-3), and three screws (SH-1-2-3)

Foam bone made of polyurethane foam 20pcf (Sawbones; Pacific Research Laboratories Inc., Vashon, WA, USA) was used as the substrate to simulate the quality of cancellous bone. Simulated acetabular cavities were prepared in the foam using hemispherical reamers of precision machinery (FANUC Robodrill; Fanuc Co., Yamanashi, Japan). The foam was mounted horizontally on a vise bench, and hemispherical-shaped domes, as acetabular cavities, were machined to a diameter of 48, 48.5, and 49 mm to simulate under-, same-size-, and over-reaming, respectively. The cups were impacted into the foam using a material-testing machine (Mini Bionix; MTS Japan Ltd, Tokyo, Japan) with a compressive force of 4,500 N and a compressive speed of 12 mm/min to ensure seating of the cup in the foam bone prior to testing. Preparation of the cavities for the screw holes was performed using a 4.5-mm-diameter manual drill, as one would use during surgery. The screws were tightened with a torque of 1.4 N m (to prevent them from spinning) in a radial direction with visual confirmation. The screws were inserted in the following six conditions with a different number and position: no screw (No-S), one screw (SH-1, SH-2), two screws (SH-1-2, SH-2-3), and three screws (SH-1-2-3) (Fig. 2).

**Fig. 3:**
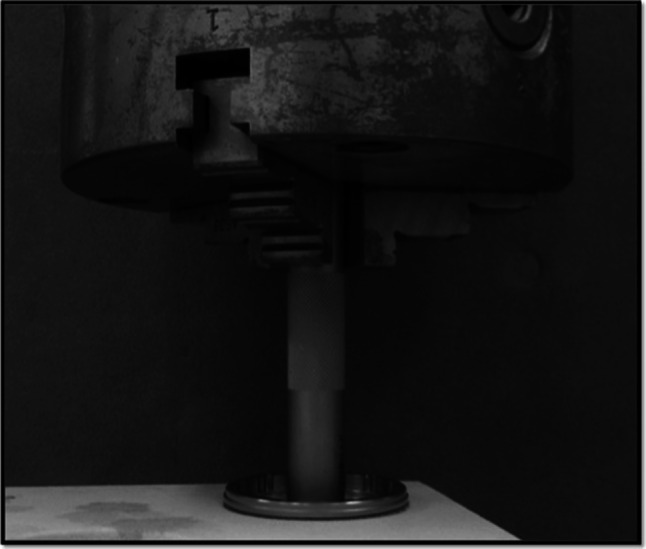
The socket is rotated by a twist-testing machine (Mini Bionix; MTS Japan Ltd, Tokyo, Japan) under a rotational speed of 0.2 rpm and a pressure of 500 N; the torque value at the start of the axial rotation between the socket and the foam bone is measured

Each specimen was mounted on a vice bench and was rotated with a twist-testing machine (Mini Bionix) under a rotational speed of 0.2 rpm and a pressure of 500 N. The torque value at the start of axial rotation between the socket and the foam bone was measured (Fig. 3). The torque value was measured three times at each of the six screw conditions in the three specimens. We calculated the effect of the additional screws on whole-cup fixation as follows: [average torque of SH-1-2-3(N m) − average torque of No-S(N m)] ÷ average torque of No-S(N m) × 100 (%). Multifactorial analysis of variance was performed to analyze the collected torque data. Using the Tukey method for multiple comparisons, the level of significance was set at 5 %.

## Results

**Fig. 4:**
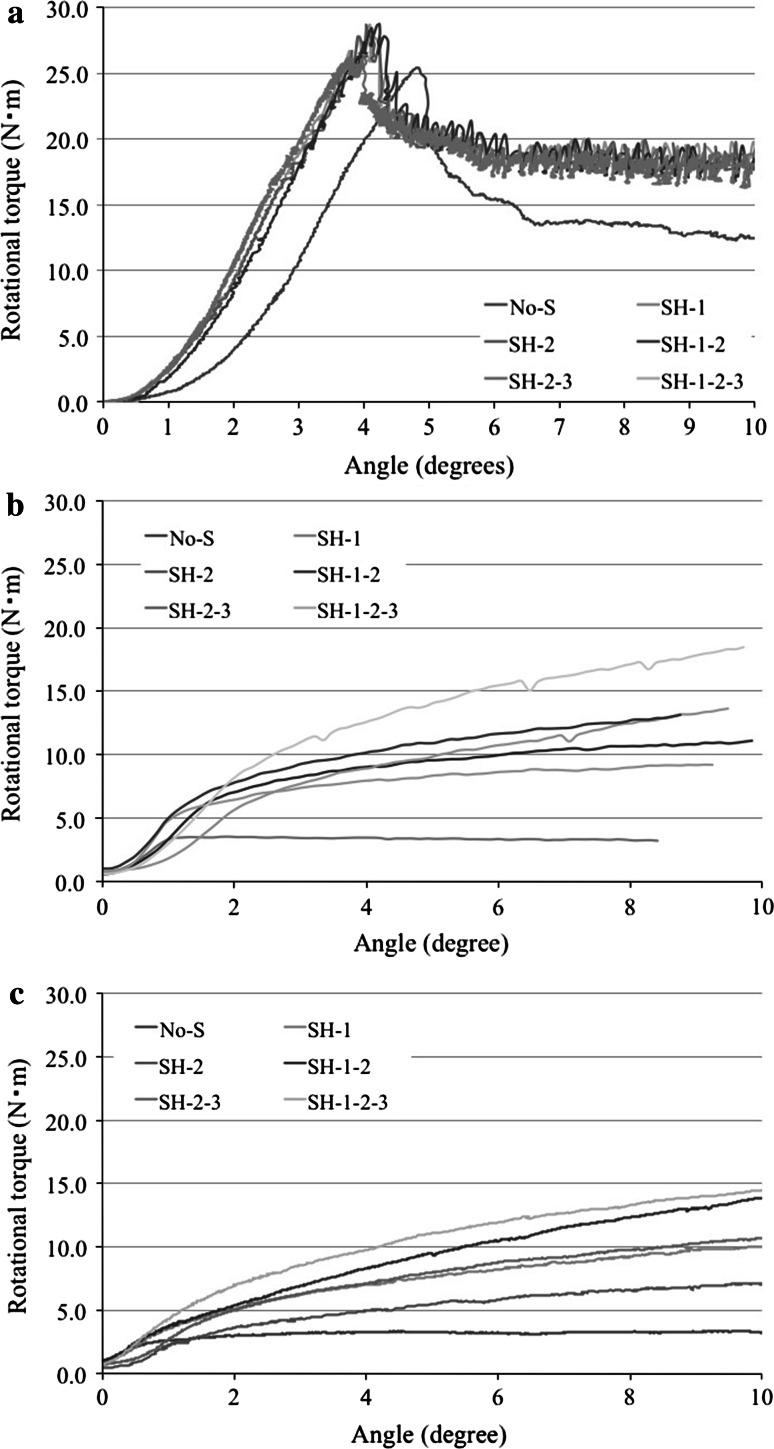
Results of the twist testing for the 48-mm-diameter reaming; the additional screw fixation is effective, but it has little influence on the whole-cup fixation (**a**). For the 48.5- (**b**) and 49-mm-diameter (**c**) reaming, the shapes of the line graphs are different from those for the 48-mm-diameter reaming, indicating marked deterioration of the cup fixation. The cup fixation tends to increase with the increasing number of screws and the addition of screws to the outer side of the cup rather than the inner side

**Fig. 5:**
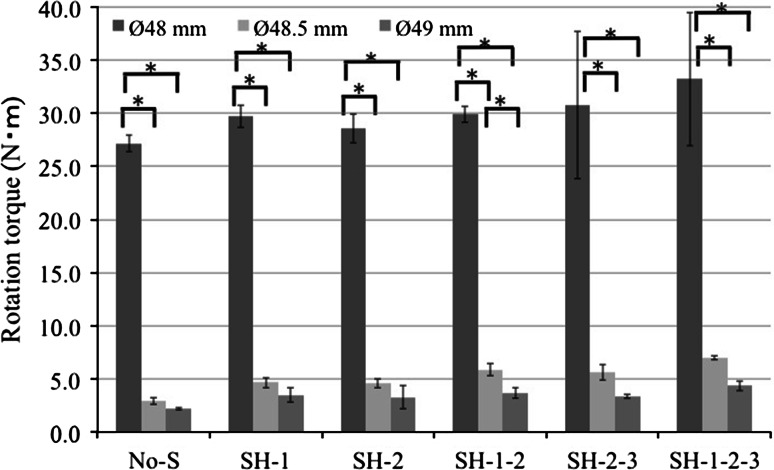
The cup fixation tends to increase with the increasing number of screws and the addition of screws to the outer side of the cup rather than the inner side. The influence of the additional screws on the whole-cup fixation is 18.1, 58.6 and 50.0 % for the 48-, 48.5-, and 49-mm-diameter reaming, respectively [(SH-1-2-3 − No-S) ÷ No-S × 100]. *P < 0.05

For the 48-mm-diameter reaming, the average maximum torques were as follow: No-S, 27.2 ± 0.8 N m; SH-1, 29.7 ± 1.1 N m; SH-2, 28.6 ± 1.4 N m; SH-1-2, 29.9 ± 0.8 N m; SH-2-3, 30.8 ± 6.9 N m; and SH-1-2-3, 33.2 ± 6.3 N m (Figs. 4a, 5). The additional screw fixation was effective, but it showed little influence on the whole-cup fixation; it was more effective with screws added to the outer side of the cup rather than the inner side. However, there were no significant differences among the screw conditions. For the 48.5-mm-diameter reaming, the average maximum torques were follows: No-S, 2.9 ± 0.3 N m; SH-1, 4.6 ± 0.5 N m; SH-2, 4.5 ± 0.4 N m; SH-1-2, 5.8 ± 0.6 N m; SH-2-3, 5.6 ± 0.8 N m; and SH-1-2-3, 7.0 ± 0.2 N m (Figs. 4b, 5). There were significant differences between No-S and SH-1-2, SH-2-3, SH-1-2-3 *P*; SH-1 and SH-1-2-3; and SH-2 and SH-1-2-3 (all, *P* < 0.05). For the 49-mm-diameter reaming, the average maximum torques as follows: No-S, 2.2 ± 0.1 N m; SH-1, 3.5 ± 0.7 N m; SH-2, 3.3 ± 1.1 N m; SH-1-2, 3.7 ± 0.5 N m; SH-2-3, 3.4 ± 0.2 N m; and SH-1-2-3, 4.4 ± 0.5 N m (Figs. 4c, 5). There were no significant differences among these screw conditions. However, in each screw conditions, there were significant differences among the 48-, 48.5-, and 49-mm-diameter reaming (*P* < 0.05) (Fig. 5). In the case of SH-1-2, there were only significant differences between the 48.5- and 49-mm-diameter reaming (*P* < 0.05) (Fig. 5). The shapes of the graphs were different from those of under-reaming, indicating marked deterioration of the cup fixation. The cup fixation tended to increase with the increasing number of screws and with addition of screws to the outer side of the cup rather than the inner side. The influence of additional screws on whole-cup fixation was 18.1, 58.6 and 50.0 %, for the 48-, 48.5- and 49-mm-diameter reaming, respectively (Fig. 5).

## Discussion

Initial cup fixation in cementless THA affects not only immediate complications, such as implant loosening during or after surgery, but also long-term outcomes. Engh et al. [5] reported that the 15-year survivorship of porous-coated acetabular cups is 94.7 ± 3.4 % for spiked cups, 98.4 ± 1.9 % for press-fit cups with screws, and 100 ± 0.1 % for press-fit-only cups. Good initial cup fixation involves both broad contact and sufficient strength between the cup and the acetabular bone bed; these factors not only contribute to good biologic fixation, but they also influence the hip center or cup coverage of the acetabular bone.

Press-fit fixation can potentially generate gaps between the cup and the acetabular bone. Gap formation or micromotion between the cup and acetabulum would likely worsen long-term outcomes. Gap formation is disadvantageous to kinematics because the hip center is lateralized more than expected, and since the cup does not directly contact the bone, widespread osseointegration can occur. Pilliar et al. [11] reported that implant displacement ≥150 μm will prevent bone formation within porous-surfaced implants and will allow attachment via well-organized fibrous connective tissue. Carlsson et al. [12] reported that a gap ≥0.35 mm at the insertion between the bone and implant will prevent direct cortical bone apposition on the implant.

However, supplemental screw fixation has a risk of vascular injury as well as particulate wear debris from the backside of the cup. Krieg et al. [13] reported the rate of backside volumetric change to be 2.8 % of the rate of volumetric articular wear. Moreover, migrated acetabular components showed significantly higher rates of backside volumetric change plus screw head indentations compared with components without migration. Hwang et al. [14] reported a case of massive retroperitoneal hemorrhage due to perforation of the external iliac vein by a drill tip passing through the anterosuperior quadrant of the acetabulum during cementless THA.

Since each technique has certain risks, it is important to be able to apply both techniques together in clinical use. In the history of cementless THA, both techniques have been used for a long time. However, clinical studies using each technique for initial fixation have not reported the long-term results or how the parameters influence one another.

Regarding the press-fit technique in cementless THA, Kaneko et al. [2] reported good clinical results and roentgenogram evaluations for 30 uncemented acetabular cups with 2-mm under-reaming, as well as good stability on biomechanical compression load and twist testing. Ng et al. [4] reported the 2- to 6-year results of 74 THAs using a cementless acetabular component without holes for supplemental screw fixation and the immediate full-weight-bearing walking allowed postoperatively. There was no migration of the acetabular components, and the majority of gaps had disappeared. Takao et al. [6] reported the 6- to 11-year results of 98 primary THAs using hemispherical porous-coated cups implanted into dysplastic hips using the press-fit technique without screws. Bone-cup contact >8.4° of the cup-center-edge angle was large enough for the cups to resist superior directed loads during the follow-up period.

Regarding screw fixation, Petersen et al. [9] reported on 324 consecutive primary THAs using a cementless cup that was reamed in a so-called line-to-line manner and fixed with 1–4 screws. The overall rate of aseptic loosening was 1 % (4 of 324) after an intermediate (10 years) follow-up. Clohisy et al. [10] reported on 196 hips in 177 patients who underwent primary acetabular reconstruction using a cementless acetabular component and screw fixation. Fixation and clinical result were deemed excellent for most patients at an average of 10 years postoperatively.

Some experimental studies have also been conducted. Kuhn et al. [15] studied the stability of cement-free press-fit acetabular cups in an in vitro displacement study. Lever-out forces increased with enhanced press-fit fixation and 1- to 2-mm under-reaming. The quality of bone and accurate cup insertion are also very important parameters in press-fit fixation. Wetzel et al. [16] studied the initial stability of press-fit acetabular cups using in vitro mechanical lever-out tests and found good results for the lever-out forces ranging 39.2–50.8 N m. They concluded that intraoperative primary stability was influenced by the quality of bone, the exactness of the reaming procedure, and the accurate cup insertion. Hsu et al. [7, 17] reported that for hemispherical cups fixed into blocks of foam bone with 0–3 screws, increasing the number of screws enhanced cup stability in the case of ideal screwing (i.e., with no eccentricity). In contrast, screw eccentricity not only reduces cup stability, but also promotes osteolysis. In their other study, using three-dimensional finite element models of the pelvis and acetabular components, screw insertion only reduced relative micromotion locally. Kwong et al. [8] confirmed that a distinct benefit of press-fit reaming is better for peripheral stability in most primary cases. In revision cases, if the bone stock is fragile, they recommended reaming line-to-line using screws. With more robust bone, they recommended trying to achieve a press-fit fixation and adding screws.

Initial cup stability in cementless THA is affected by many factors including bone quality and diameter and the design and surface treatment of the cup. However, it is certain that press-fit and supplemental screw fixation is a principal technique for obtaining good initial fixation of a cementless cup. In actual surgery, these two techniques do not conflict with one another, and they can be performed as a set. Thus, it is of clinical importance to reveal the influence of these techniques on initial fixation. Our study demonstrated that press-fit fixation obtained greater fixation strength than additional screw fixation. According to our experiment, press-fit fixation of a cementless cup achieved rigid stability, since a reamer’s margin of only 0.5 mm led to a difference in outcome. However, in actual surgery, it is difficult to perform precise reaming of the acetabulum especially with insufficient bone due to osteoporosis. Although the addition of screws increased stability of the implant under good press-fit conditions, this study demonstrated that additional screws had little impact on whole-cup stability. Roth et al. [3] compared 101 cementless cups implanted by press-fit fixation without the use of screws and 110 cementless cups with additional fixation with 1–3 screws at the upper part of the acetabulum. They concluded that additional screw fixation is not vital if good press-fit fixation is obtained, since press-fit-only cementless cups lessen radiographic change around the cup and do not have any clinical disadvantage.

In cases without adequate press-fit fixation, cup stability depends on the strength of screw fixation. In this study, increasing the number of screws resulted in increased cup stability. Screw insertion apart from the cup center was found to be more effective for the rotation torque of the cup compared with screw insertion close to the cup center. Some caution should be noted with this technique, such as in cases with dysplastic osteoarthritis, which leads to insufficient contact between the cup and acetabular bone. However, the surgery should still be performed in the light of this risk. When comparing the 48.5- and 49-mm reaming, this study revealed that in the near-reaming size to press-fit fixation, screw fixation was more effective in increasing cup stability. This finding also suggests the importance of reaming the acetabulum precisely.

It is interesting how much initial cup fixation strength is required clinically. Most patients will be allowed to perform early full-weight bearing and running after surgery. Kanda et al. [18] calculated the initial fixation power as: *T* = *R* × *f* × *L*, in which *T* is the theoretical rotation torque, *R* is the femoral head radius, *f* is the coefficient of friction, and *L* is the load. We assumed up to 6-times the weight in consideration of safety. Concerning the polyethylene liner and metal femoral head, the coefficient of friction (*f*) was 0.1–0.2 and the femoral head radius (*R*) was 16 mm. With a body weight of 60 kg, as the average Japanese weight, the load (*L*) was indicated as follows: 60 kg × 9.8 N = 588 N kg. Thus, the rotation torque, which occurs in the cup with postoperative normal gait, was expressed by the following numerical formula: *T* = 0.016 × (0.1 − 0.2) × (588 × 6). Therefore, the theoretical rotation torque was 5.64–11.29 N m. In the 48.5-mm-diameter reaming, >3 additional screws exceeded the theoretical rotation torque. In the 49-mm-diameter reaming, no number of additional screws were able to exceed the theoretical rotation torque.

Limitations of this study include the use of Sawbones instead of human bone, which have differences in quality. In addition, the density and geometry of the Sawbones, surface treatment of the cup, length and orientation of the screws, and addition of >4 screws were not assessed in this study. Although each factor would affect the experimental results, they would not greatly affect the overall premise of the study. It is the decision of each surgeon to determine whether initial fixation is appropriate in daily practice, since there is no good specific method for measuring it.

In conclusion, the press-fit technique was extremely effective for gaining sufficient initial cup fixation. Additional screw fixation was effective under good press-fit conditions, but it showed little impact on whole-cup stability. When adequate press-fit fixation cannot be achieved, cup stability decreases considerably and depends on the strength of screw fixation, which is influenced by the length and position of the screws. However, screw fixation was found to be more effective for increasing the cup stability strength in the same-size reaming than in over-reaming in press-fit fixation compared with under-reaming. Thus, surgeons must perform reaming of the acetabulum with high precision in order to gain sufficient press-fit fixation in cementless THA. If unable to achieve an adequate press-fit fixation, screws should be inserted cautiously with regard to their number and placement.
